# A new sensitizer DVDMS combined with multiple focused ultrasound treatments: an effective antitumor strategy

**DOI:** 10.1038/srep17485

**Published:** 2015-12-03

**Authors:** Wenli Xiong, Pan Wang, Jianmin Hu, Yali Jia, Lijie Wu, Xiyang Chen, Quanhong Liu, Xiaobing Wang

**Affiliations:** 1 Key Laboratory of Medicinal Resources and Natural Pharmaceutical Chemistry, Ministry of Education, National Engineering Laboratory for Resource Developing of Endangered Chinese Crude Drugs in Northwest of China, College of Life Sciences, Shaanxi Normal University, Xi’an 710062, Shaanxi, China

## Abstract

Sonodynamic therapy (SDT) was developed as a promising noninvasive approach. The present study investigated the antitumor effect of a new sensitizer (sinoporphyrin sodium, referred to as DVDMS) combined with multiple ultrasound treatments on sarcoma 180 both *in vitro* and *in vivo*. The combined treatment significantly suppressed cell viability, potentiated apoptosis, and markedly inhibited angiogenesis *in vivo*. *In vivo*, the tumor weight inhibition ratio reached 89.82% fifteen days after three sonication treatments plus DVDMS. This effect was stronger than one ultrasound alone (32.56%) and than one round of sonication plus DVDMS (59.33%). DVDMS combined with multiple focused ultrasound treatments initiated tumor tissue destruction, induced cancer cell apoptosis, inhibited tumor angiogenesis, suppressed cancer cell proliferation, and decreased VEGF and PCNA expression levels. Moreover, the treatment did not show obvious signs of side effects or induce a drop in body weight. These results indicated that DVDMS combined with multiple focused ultrasounds may be a promising strategy against solid tumor.

Photodynamic therapy (PDT) and sonodynamic therapy (SDT) were developed as novel auxiliary therapeutic strategies for the management of solid tumors^1^,^2^,^3^ following surgery, radiotherapy and chemotherapy. PDT has advantages over surgery or radiotherapy because it reduces long-term morbidity and permits the selection of alternative treatments in cases of recurrent or residual disease, or a second primary disease^4^. Although PDT has been clinically applied to treat many types of cancer (e.g., esophageal, lung, bladder, and cervical cancer^5^,^6^,^7^,^8^), it has two notable shortcomings: limited penetration of light into deep tumor tissue, which is required to activate the photosensitizer, and result in potentially serious side effects, such as long-lasting skin sensitivity due to the retention of the photosensitizer in cutaneous tissues^9^,^10^.

Sonodynamic therapy (SDT) was established in 1989 and was developed as a promising noninvasive approach based on PDT^11^,^12^; it has been widely studied in cancer cells *in vitro*^13^,^14^,^15^ and in animals bearing malignant tumors^16^,^17^. Unlike visible light, ultrasound is a type of mechanical wave that can penetrate a cancer target buried deep within human tissue. Therefore, SDT overcomes the major limitation of PDT. Moreover, ultrasonic therapy can be repeated in the clinic because it is relatively safe, easily accessible and non-toxic. Recently, ultrasound has become a preferred clinical technique for the regulation of targeted therapy^18^,^19^,^20^.

Owing to the rapid expansion of knowledge on the fundamental mechanisms underlying SDT, investigators have recognized that the sonosensitizer is one of the most essential factors in SDT. The discovery and development of new more efficient sonosensitizers is the major focus in this field. Recently, a novel sensitizer (sinoporphyrin sodium, referred to as DVDMS) was granted independent intellectual property status in China^21^. This sensitizer shows higher photochemical activity than Photofrin®, which has been approved by the FDA for use as a sensitizer in PDT for cancer^22^. Moreover, our previous studies showed that DVDMS could be efficiently activated by ultrasound and exhibited significant tumoricidal activity both *in vivo* and *in vitro*^21^,^23^. *In vivo*, the tumor weight inhibition ratio was 55.37% when the tumor was exposed to ultrasound 2 h after DVDMS (2 mg/kg) administration, suggesting that DVDMS might be a potential sonosensitizer. Moreover, DVDMS is a purified and well-defined sensitizer rather than a complex mixture^21^.

We found that the biodistribution of DVDMS in tumors reached a peak at 2 h post-injection, using an IVIS spectrum small animal imaging system *in vivo*^23^. Additionally, the fluorescence signal in the tumor was several times higher than the signal in most normal tissues. Thus, 2 h after DVDMS administration might be an appropriate time point for ultrasound exposure to achieve a therapeutic effect on tumors (high concentration in tumors) and to minimize side effects on healthy tissues (low concentration in surrounding healthy tissues). Moreover, we found that the DVDMS concentrations at 6 h and 24 h reached approximately 98.77% and 70.37% of the maximum concentration in the tumor, respectively. Therefore, it is possible that multiple ultrasound exposures within 24 h after injection may enhance the treatment efficacy. Here, we focused our investigation on the anti-tumor effects of the new sensitizer DVDMS combined with multiple ultrasounds by examining apoptosis, angiogenesis and the safety of the agent *in vitro* and *in vivo*. To the best of our knowledge, the present study is the first *in vitro* and *in vivo* preclinical study to assess the anti-tumor effects of multiple ultrasounds combined with DVDMS. These findings may have important implications for the treatment of cancer.

## Results

### Cellular uptake of DVDMS

**Figure 1:**
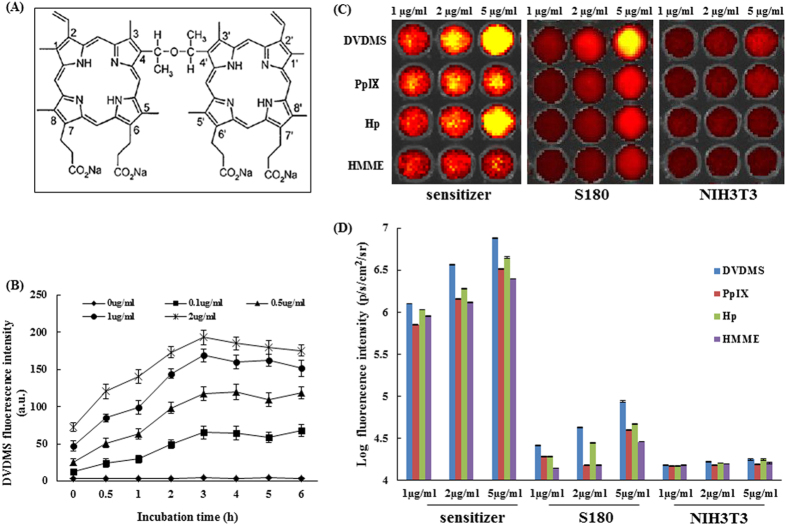
Fluorescence intensity of different sensitizers in normal cells and cancerous cells under the same experimental conditions. (**A**) Chemical structure of DVDMS. (**B**) Cellular uptake of DVDMS. (**C**) Fluorescence photographs of the four sensitizers, sensitizers in NIH3T3 cells and S180 cells in NIH3T3 cells and S180 cells under the same experimental conditions. (**D**) Fluorescence intensity of (**C**). The fluorescence intensity was recorded as photons per second per centimeter squared per steradian (p/s/cm^2^/sr). Data are presented as the mean ± standard deviation of three independent experiments.

The optimum uptake of DVDMS by 180 cells was investigated prior to ultrasonic irradiation. The uptake kinetics were determined by measuring the cellular fluorescence signal using flow cytometry. As shown in Fig. 1B, the fluorescence signal of DVDMS elevated gradually and reached a maximum at approximately 3 h when different concentrations were used. Thus, in the following experiments, we chose 3 h as the incubation time of DVDMS with cells.

### Cellular uptake of four types of porphyrin sensitizers

The intensity of different sensitizers in S180 (cancerous) cells and NIH3T3 (normal) cells was analyzed using fluorescence imaging. As shown in Fig. 1C, treatment with 5 μg/mL of DVDMS, compared with other sensitizers, resulted in brighter florescence in both cancerous cells and normal cells. Notably, the florescence intensity in the cancer cells was much higher than in the normal cells. Further testing clearly demonstrated that the florescence intensity of DVDMS (Fig. 1D) was significantly higher in the S180 cells, suggesting that DVDMS, compared with the other three sensitizers, accumulated to a much larger extent in cancer cells.

### Cell viability assessment

**Figure 2:**
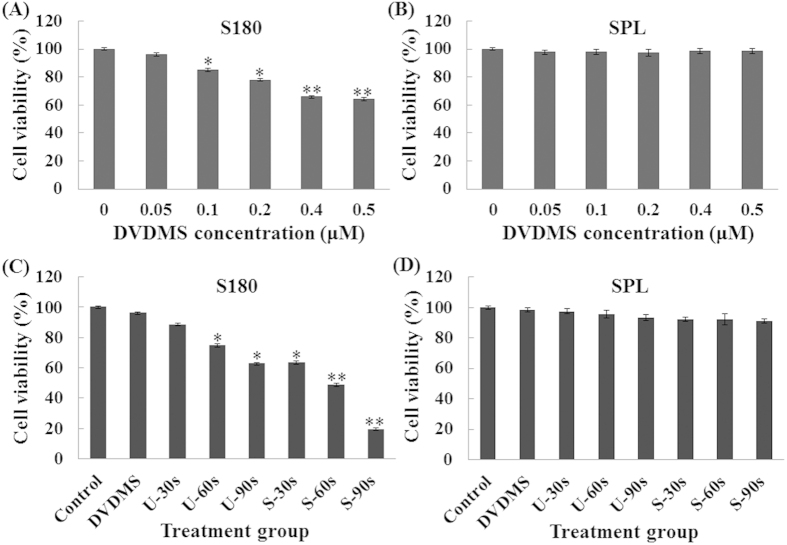
Cytotoxicity studies of DVDMS. Cell viability was measured by the MTT assay. After 3 h of incubation, the cells were exposed to different concentrations of DVDMS and the viability of tumor cells [(**A**) S180] and normal cells [(**B**) SPL] were measured. The cytotoxicity of DVDMS-mediated sonodynamic therapy and the viability of tumor cells [(**C**) S180] and normal cells [(**D**) SPL] were measured. Data are means ± SD of three independent experiments. *p < 0.05 and **p < 0.01 versus the concentration of DVDMS at 0 μM. MTT, 3-(4, 5-dimethylthiazol-2-yl)-2, 5-diphenyltetrazolium bromide.

As shown in Fig. 2A, DVDMS inhibited the cell viability of S180 cells in a dose-dependent manner. A significant loss in cell viability was observed when the cells were incubated with 0.1 μM DVDMS (84.98%). The cell damage caused by ultrasound alone increased with the irradiation time. The inhibition rate was significantly increased when the cells were treated with DVDMS combined with ultrasound. In the presence of 0.05 μM DVDMS, the cell viability decreased to 63.54%, 48.79%, and 19.55% with ultrasound irradiation times of 30, 60, and 90 s, respectively (Fig. 2C).

We also investigated the viability of SPL (spleen lymphocytes) after SDT mediated by 0.05 μM DVDMS. Interestingly, in contrast with the tumor cells, no significant changes were observed in the viability of normal cells (Fig. 2B,D). On the basis of these findings, we suggest that DVDMS can selectively kill tumor cells; this effect may be related to its preferential accumulation in tumor cells.

### Detection of cell apoptosis using flow cytometry

**Figure 3:**
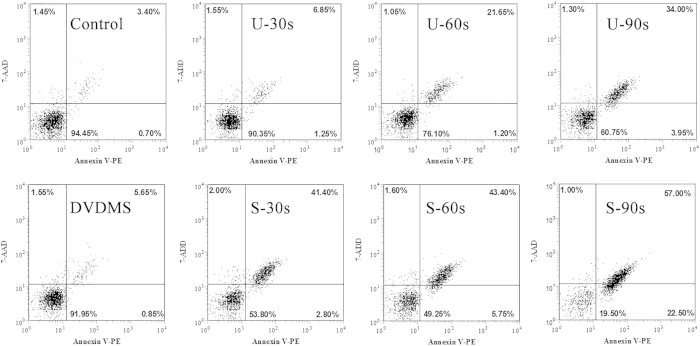
Induction of apoptosis after different treatments in S180 cells. Dot plot graphs show viable cells (AV^−^/7AAD^−^), early apoptotic cells (AV^+^/7AAD^−^), late apoptotic cells (AV^+^/7AAD^+^) and necrotic cells (AV^−^/7AAD^+^).

Apoptotic rates were measured by Annexin V-PE and 7-ADD staining after treatment. Figure 3 showed that there were 94.45% viable cells and 4.10% apoptotic cells in the control group. In the U-30s (ultrasound alone-30s) and DVDMS groups (0.05 μM DVDMS alone), the proportion of viable cells decreased to 90.35% and 91.95%, respectively. When the cells were treated with SDT, the viability in groups S-30s (sonication-30s plus 0.05 DVDMS), S-60s (sonication-60s plus 0.05 DVDMS), and S-90s (sonication-90s plus 0.05 DVDMS) decreased to 53.81%, 49.25%, and 19.50% and the proportion of apoptotic cells increased to 44.20%, 49.15%, and 79.50%, respectively.

### DVDMS-mediated SDT inhibits capillary-like structure formation of endothelial cells

**Figure 4:**
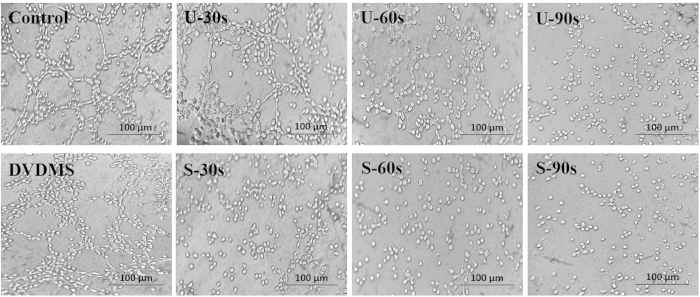
Sonodynamic therapy inhibits capillary-like structure formation in endothelial cells. HUVECs stimulated with 10 ng/mL VEGF were allowed to spontaneously form tube networks after different treatments. The tubular networks were visualized and photographed after 4–6 h.

Although angiogenesis is a complex process, tube formation is one of the key steps. We evaluated the ability of HUVECs to form tube-like structures on Matrigel after treatment with DVDMS-mediated SDT. When HUVECs were placed on the growth factor–reduced Matrigel, elongated, cross-linked and robust tube-like structures were formed in the presence of VEGF. As shown in Fig. 4, control group cells induced robust tube formation; DVDMS treatment of the cells did not cause a significant reduction in the tube network. However, a significant reduction in the tube network was observed for U-90s (ultrasound alone-90s) cells and S-90s cells compared with control cells, resulting in a decline in the number, length, and area of the capillary-like structures. These results indicated that tube formation was markedly inhibited by multiple ultrasonic treatments.

### Tumor growth is significantly inhibited by DVDMS-SDT

**Figure 5:**
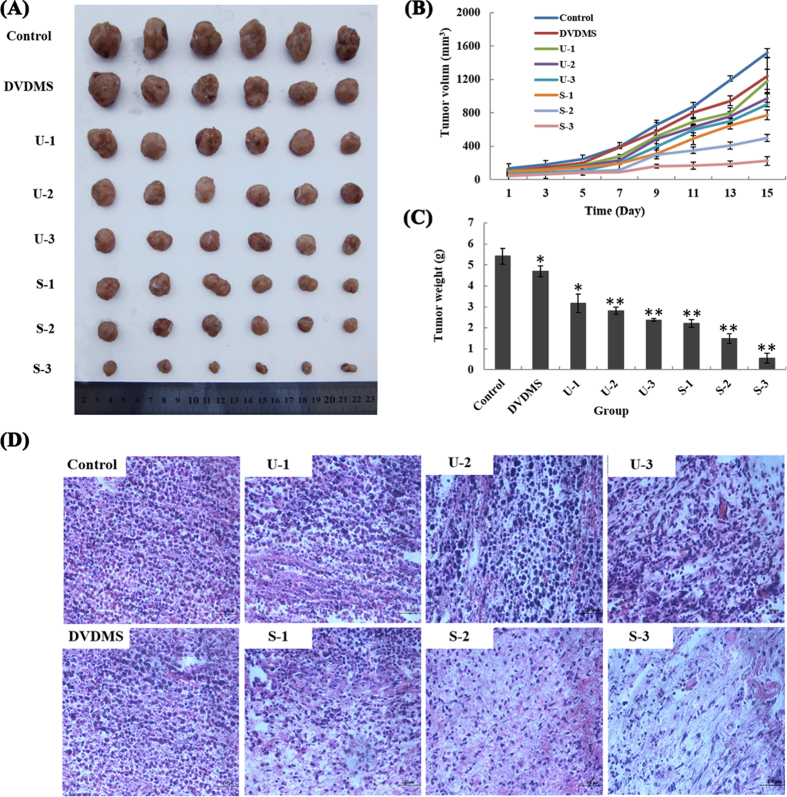
Antitumor effect of DVDMS combined with multiple focused ultrasound treatments *in vivo*. (**A**) Gross morphology of tumors excised from each group of mice 15 days after treatment. (**B**) The development of tumor volume after various treatments. (**C**) Tumor weight in each group on the 15th day after treatment. The results represent the mean ± SD. *p < 0.05, **p < 0.01 versus the control. (**D**) Morphological changes in H&E-stained tumor sections.

The anti-tumor activity of DVDMS-mediated SDT *in vivo* was determined by examining tumor growth (volume and weight) changes in S180 xenografted mice. Our results showed that the administration of multiple sonodynamic therapies significantly decreased the tumor growth rates of the xenografts (Fig. 5A–C and supplementary materials Table 1). The tumor volume remained nearly unchanged when the tumors were treated with DVDMS alone compared with control. Ultrasound alone (U-1, ultrasound alone-once) resulted in some degree of anti-tumor activity. The anti-tumor activity became stronger when the number of ultrasound treatment increased (ultrasound alone-twice (U-2) and ultrasound alone-thrice (U-3) groups). Ultrasound combined with DVDMS substantially inhibited tumor growth, and the effect was noticeably enhanced with an increase in ultrasound treatment times. The effect of S-3 (sonication-thrice plus DVDMS) group therapy was quite durable because it was maintained throughout the therapy period.

Supplementary materials Table 1 summarized the tumor weight inhibition by the different protocols on day 15 post-treatment. The tumor weight inhibition ratios were approximately 19.71% (P < 0.05) and 32.56% (P < 0.05) for DVDMS and U-1, respectively. In contrast, the tumor weight inhibition ratio was 89.82% (P < 0.01) for S-3 group, indicating that DVDMS repressed tumor growth and that the effect was strengthened with the increase in ultrasound irradiation time. This finding is consistent with the tumor volume results.

### Histopathological changes in tumor sections

After paraffin sectioning, the tumor tissues of different groups were observed under a light microscopy after H&E staining (Fig. 5D). Histological examination of the untreated tissue showed compact tumor cells with intact structures. In contrast, the tumor tissues were sparse and separated from each other in the ultrasound alone groups (U-1, U-2 and U-3). The structure of the tumor tissue was more seriously damaged in the SDT groups (S-1 (sonication-once plus DVDMS), S-2 (sonication-twice plus DVDMS) and S-3) than in ultrasound alone groups and was accompanied by obvious vacant sections and a large amount of nuclear fragments.

### Apoptosis *in vivo*

**Figure 6:**
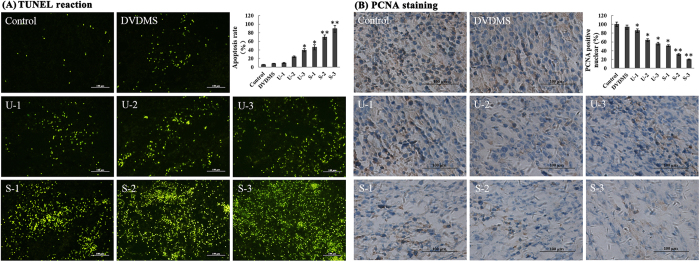
Proliferation of S180 cells assessed with immunohistochemical staining against PCNA and the TUNEL assay. (**A**) Immuno-histochemical staining of PCNA in tumors. (**B**) Tumor sections were stained using the TUNEL method. Quantitative number of PCNA- positive and TUNEL- positive cells were determined by manually counting six random fields for each group. The results represent the mean ± SD. *p < 0.05, **p < 0.01 versus the control.

TUNEL assays combined with fluorescence microscopy were used to detect whether apoptosis could be induced by DVDMS-mediated SDT *in vivo*. As show in Fig. 6A, one round of ultrasound alone (U-1) resulted in less apoptosis, whereas positive particles exhibiting green fluorescence (indicative of apoptosis) were significantly increased in the corresponding S-1 group. The nuclei of some tumor cells contained chromatin and were fragmented, demonstrating typical features of apoptosis. The apoptosis rate increased with the number of ultrasounds (one, two, and three) in the sonication plus DVDMS groups and was at least 4.7-, 7.2-, and 9.3-fold greater, respectively, than in the group that received a single ultrasound alone (U-1). The data indicated that DVDMS-mediated SDT could also induce apoptosis *in vivo*, thereby contributing to tumor growth suppression. This phenomenon may represent the primary mechanism underlying the arrest of cancer cell proliferation.

To investigate the anti-proliferative activity of DVDMS-mediated SDT on sarcoma tumors *in vivo*, we directly analyzed the expression of PCNA protein by immunohistochemistry (Fig. 6B). Therapy with three dose of ultrasound alone (U-3), compare to the vehicle treatment, led to a 56.12% reduction in PCNA-positive cells in the S180 tumors, whereas a reduction in PCNA expression of 20.62% was observed in the S-3 treatment group. These results suggest a potent and cooperative anticancer action of DVDMS-mediated SDT in the tumor xenograft model.

### DVDMS-mediated SDT inhibits tumor angiogenesis *in vivo*

**Figure 7:**
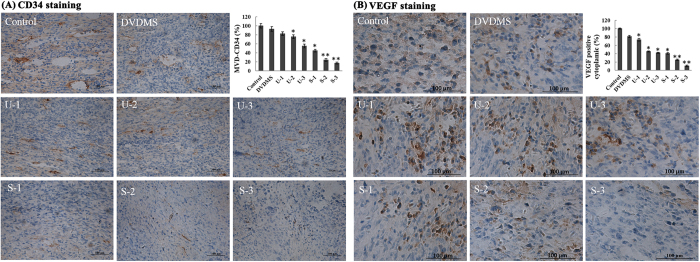
Inhibition of angiogenesis and VEGF levels detected by immunohistochemistry. (**A**) Paraffin sections of S180 tumors were tested by immunohistochemistry analysis with an anti-CD34 antibody. Representative images of the tumor vasculature from vehicle and differently treated mice were shown. (**B**) The VEGF level after different treatments. For VEGF analysis, 6 areas were randomly selected under a microscope. Image Pro Plus 6.0 (Media Cybernetics, Inc., Bethesda, MD, USA) was used to quantify the extent of immunopositive expression in cells with integrated optical density (IOD) values. The fields were averaged for each tumor, and the averages for each animal were used to calculate the final mean ± SD, *p < 0.05, **p < 0.01.

Anti-CD34 immunohistochemical analysis of tumor sections from the S180-xenografted mice revealed that DVDMS-mediated SDT significantly reduced microvessel density (MVD) after treatment. As shown in Fig. 7A, DVDMS-SDT, compared with ultrasound therapy alone, resulted in enhanced inhibition of the tumor vasculature indicated by CD34-positive endothelial cells (brown). The data revealed that DVDMS-mediated SDT inhibited tumor angiogenesis *in vivo*, which aided in the suppression of tumor growth.

### DVDMS-SDT inhibited VEGF expression

The induction of new blood vessel formation by tumor cells (tumor angiogenesis) is closely associated with VEGF production. As shown in Fig. 7B, the VEGF levels were high in the control and DVDMS groups. In contrast, ultrasound decreased VEGF expression in a treatment time-dependent manner; furthermore, its combination with DVDMS further decreased VEGF expression, which was consistent with their inhibitory effects on CD34 expression.

### Safety profile of DVDMS-mediated SDT

**Figure 8:**
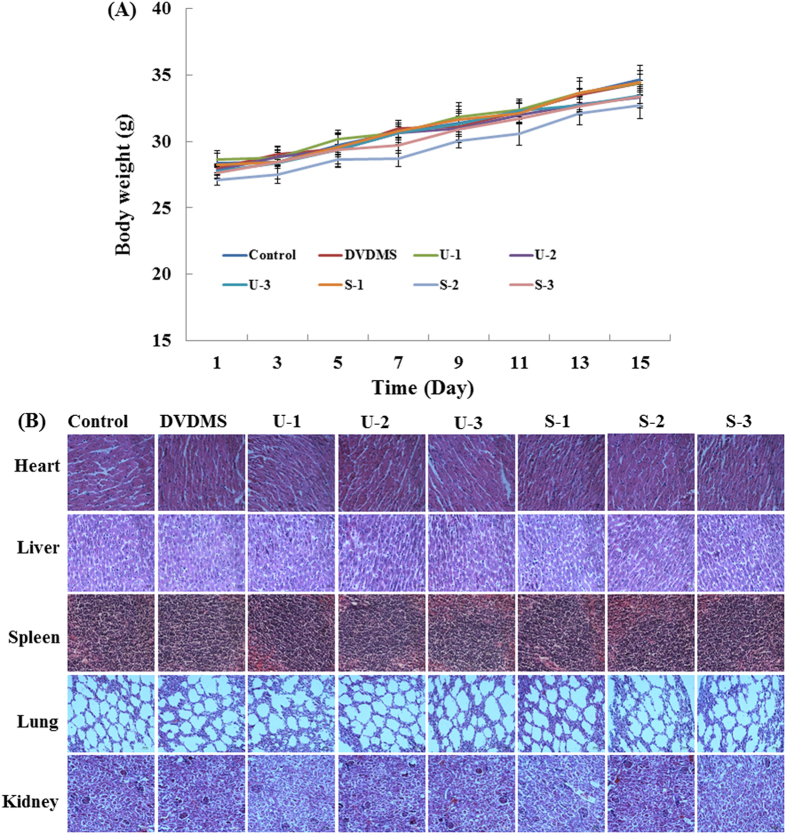
Safety profile of DVDMS-mediated SDT. (**A**) Body weight curve of S180 tumor -bearing mice after treatment. (**B**) H&E stained images of liver, spleen, kidney, heart and lung.

In the acute toxicity test, mortality, clinical signs, and body weight (Fig. 8A) of the mice were measured for 15 days; no obvious changes were observed. Based on this result, the No Observed Adverse Effect Level (NOAEL) of DVDMS-mediated SDT could be 2 mg/kg. During the treatment of mouse xenografts in ICR mice, we did not observe obvious adverse effects, such as skin sensitivity, diarrhea or toxic death, in the treated group. Organ coefficients of the mice did not show any pathological changes (supplementary materials Table 2). Moreover, microscopic examination of the heart, liver, spleen, lung, and kidney revealed no pathological changes after DVDMS-mediated SDT treatment compared with the vehicle treatment group (Fig. 8B).

## Discussion

Although targeted therapy for cancer has advanced significantly, cancer remains one of the leading causes of mortality worldwide. Conventional treatments such as surgery, chemotherapy, and radiation suffer from numerous shortcomings, such as systemic toxicity, low selectivity, drug resistance and potential long-term side effects^24^. Therefore, the development of highly selective and minimally invasive cancer treatments is urgently needed. SDT with low-intensity ultrasound combined with a sonosensitizer may represent a promising approach to cancer therapy. The use of ultrasound has the advantages of being noninvasive and convenient while also possessing deep-penetration properties.

Some researchers have argued that ultrasound may promote the spread of tumor cells and accelerate metastasis^25^; however, we did not observe tumor spread to other tissues or metastasis to other organs when we stripped away the tumors. Likewise, no significant increase in the number of lung metastases was observed in a preclinical study of ablative exposure to a highly metastatic prostate cancer line implanted into the hind legs of mice^26^ or in the number of lung metastases observed in a study of high-amplitude ultrasound exposure on subcutaneous tumors implanted into the hind legs of mice^27^. These findings lead us to hypothesize that the emergence of spread may be related to ultrasound intensity. Ultrasound-induced spread occurs only when the intensity increases beyond the threshold of cavitation. Both ultrasound (i.e., intensity, frequency, waveform, exposure duration, focal depth in tissue, and exposure methodology) and tissue (i.e., type and physiological status) characteristics affect treatment outcomes. Exposure to an identical form of ultrasound irradiation may produce drastically different effects in different tissues. Therefore, exploration of the mechanism underlying ultrasound-enhanced chemotherapy must be conducted according to the experimental conditions, and ultrasound parameters must be screened for each case to achieve the desired therapeutic effect.

On the basis of previous research, we used a strategy that combined DVDMS with multiple focused ultrasound treatments to improve the antitumor effect. Our previous work demonstrated that DVDMS exhibited characteristics such as fast tumor targeting and a sustained relatively high level out to 24 h post-injection, followed by a slight decrease over time. Therefore we implemented triple-focused ultrasound treatment from 2 to 24 h.

SDT of cancer is based on preferential uptake and/or retention of sonosensitizer in tumor tissues and subsequent activation of the drug by ultrasound irradiation, specially causing tumor killing. In the present study, multiple ultrasound combined with DVDMS displayed much more significantly tumor growth inhibition compared with single ultrasound treatment. We speculate two factors may contribute a lot: the accumulation content of sensitizer in tumor tissue, ultrasound parameters (ultrasound intensity, frequency, exposure time etc.). The optimum parameters greatly affect SDT efficacy. In addition, appropriate time windows for ultrasound exposure should be chosen when the sensitizer is highly accumulated in tumors. We know, as the ultrasound exposure duration goes on, the content of sensitizer declines in tumor. When the sensitizer is consumed to a certain degree, the combined effect will vanish just representing the ultrasound alone effect. Previous study indicated DVDMS in tumors reached a peak at 2 h post-injection, DVDMS concentrations at 6 h and 24 h of DVDMS were about 98.77% and 70.37% of the peak, respectively23. This study utilizes three times of ultrasound exposure at 2, 6 and 24 h after DVDMS injection to maximumly excite DVDMS in the tumor tissue, producing much higher anti-tumor efficiency.

Acoustic cavitation is known to be the primary cause of sonoluminescence, mechanical shock waves and sonochemical reaction producing reactive oxygen species. The mechanical shock waves and reactive oxygen can destroy tumor cells. Maybe, a single treatment with longer time could also lead to the same ablation. What we need to be cautious is that the mice cannot tolerate the exposure time beyond some threshold. If the treatment time extends, thermal effect would damage normal surrounding tissues. In our pre-test, we found mice death occurred (3 of 10 mice died in the therapeutic process) when the treatment time was over six minutes. By full consideration, we adopt intermittent multiple ultrasound treatment strategy in the current study to improve SDT efficacy and simultaneously avoid harmful effects.

Given that DVDMS is a novel sensitizer, we initially compared DVDMS with other porphyrin sensitizers in both normal and cancerous cells. A selective accumulation and cytotoxicity of DVDMS in tumor cells were shown. The ability of SDT to induce cellular apoptosis has been confirmed in several cell lines^15^,^28^,^29^,^30^ and is regarded as the primary mechanism by which SDT inhibits tumor growth. Our results both *in vitro* and *in vivo* suggested that under the given experimental conditions, the combination of DVDMS and multiple ultrasound application significantly increased the number of apoptotic cells and reduced tumor growth. The tumor weight inhibition ratio approximately improved by 1.6 fold compared to that of our previous study with the same DVDMS dose and single ultrasound treatment.

Proliferating cell nuclear antigen (PCNA) is an essential organizer “hub” protein that is involved in DNA replication and repair^31^. Recently, studies have revealed that PCNA is also linked to various cytosolic functions, such as the regulation of apoptosis, metabolism, and antitumor immunity^32^,^33^,^34^. Here, we monitored a significant PCNA drop after DVDMS plus multiple ultrasound treatments compared with either mono-treatment, indicating that disaggregation of PCNA may contribute to tumor cell apoptosis.

Angiogenesis occurs via a complex step-wise process that includes proteolytic degradation of the basement membrane, the proliferation and invasion of endothelial cells, and the formation of functional capillary lumens^35^. One of the initial steps in tumor-induced angiogenesis is the secretion of multiple angiogenic factors from tumor cells, including vascular endothelial growth factor (VEGF), basic fibroblast growth factor (bFGF), and platelet-derived growth factor (PDGF). Among these, VEGF is the most important pro-angiogenic factor, and the level of VEGF is an important prognostic marker of tumor angiogenesis^36^. VEGF is now accepted to play a major role in the continuous growth and metastasis of tumors^37^. The results showed that the VEGF expression level was significantly down-regulated in the multiple treatment groups compared with the control, which was consistent well with the antiangiogenesis data of CD34.

Based on the results of *in vivo* toxicity study, DVDMS is safe at the tested dose. The inhibition of tumor growth by DVDMS-mediated SDT is not attributable to systemic toxicity. However, our evaluation of DVDMS-PDT safety was somewhat limited. It will be necessary to perform a rigorous safety assessment before this treatment is used in clinical cancer therapy.

In conclusion, the multiple ultrasound strategy is a relatively safe, repeatable, easily accessible, inexpensive, non-invasive, and non-toxic form of treatment that can be focused on a specific region deep in the tissue. Importantly, the combination of multiple ultrasounds with the new sensitizer DVDMS showed efficient anticancer effects in mouse sarcoma 180 cells *in vivo* and *in vitro*, including apoptosis induction, proliferation inhibition and suppression of tumor microvasculature formation, with no visible side effects. These results indicate that DVDMS combined with multiple ultrasounds may represent a new promising strategy against solid tumors.

## Materials and Methods

All methods were performed in accordance with the approved guidelines. The animal experiments were performed in accordance with the National Institute of Health’s Guide for the Care and Use of Laboratory Animals and were approved by the university’s Institutional Animal Care and Use Committee of Shaanxi Normal University (Xi’an, China).

### Chemicals

DVDMS (molecular formula: C_68_H_66_N_8_O_9_Na_4_, Molecular weight: 1230.265, purity > 98%) is the property of Qinglong Hi-tech Co, Ltd (Jiangxi, China) and was kindly provided by Professor Qicheng Fang from the Chinese Academy of Medical Sciences (Beijing, China). DVDMS was dissolved in phosphate-buffered saline to a stock concentration of 1 mM and stored in the dark at −20 °C. The chemical structure of DVDMS is shown in Fig. 1A. All other reagents were commercial products of analytical grade.

### Cell culture

Mouse sarcoma 180 and NIH3T3 cells were obtained from the cell bank of the Chinese Academy of Science (Shanghai, China) and cultured in RPMI 1640 medium (Gibco, Life Technologies Inc., Grand Island, NY, USA). Human umbilical vein endothelial cells (HUVECs) were a gift from the Fourth Military Medical University and were cultured in Dulbecco’s Modified Eagle’s Medium (DMEM, Gibco, Life Technologies, Inc.). All cells were cultured in media supplemented with 10% fetal bovine serum (FBS, HyClone, Logan, UT, USA), 1% penicillin–streptomycin and 1% glutamine in an incubator with 5% CO2 and 100% humidity at 37 °C.

### Isolation of normal mouse spleen lymphocytes (SPL)

In this study, the SPL isolated from healthy Institute of Cancer Research (ICR) mice (weight: 18–22 g, female, supplied by the Experimental Animal Center, Fourth Military Medical University, Xi’an, China) were used as normal cells to assess the cytotoxicity of DVDMS *in vivo*. SPL were isolated with the lymphocyte separation kit (Applygen Technologies Inc., Beijing, China) according to the manufacturer’s instructions.

### Sonication device and treatment protocol *in vivo* and *in vitro*

The *in vivo* experimental apparatus for ultrasound was similar to that previously described^23^. The frequency of the ultrasound was 1.90 MHz, and the ultrasound power could be adjusted by an amplifier (ultrasound generator, AG1020) purchased from T&C Power Conversion Inc. (Rochester, NY, USA). The load power (LP) of the AG1020 apparatus indicated as 4 W was adopted in this study. When tumors grew to approximately 5–7 mm in diameter (approximately 6 days later), the animals were randomly divided into eight groups: the control group (control), 2 mg/kg of DVDMS solution alone (DVDMS), ultrasound alone-once (U-1), ultrasound alone-twice (U-2), ultrasound alone-thrice (U-3), sonication-once plus DVDMS (S-1), sonication-twice plus DVDMS (S-2), and sonication-thrice plus DVDMS (S-3). Ultrasound radiation was applied for 3 min at each treatment. Injections were performed into the caudal vein; after DVDMS administration (2 h, 6 h and 24 h), the mice were exposed to the first, second and third sonications. Within this period of time, DVDMS was sustained at a relatively high level. All experiments were performed in the dark to avoid DVDMS excitation.

*In vitro*, the experimental set-up for insonation was the same as previously described^38^. The focused ultrasound transducer with a frequency of 1.1 MHz was submerged in degassed water in the tank facing directly upward. The LP of the amplifier indicated as 2 W was adopted in this study. The sarcoma 180 cell suspension was divided into eight groups: control (control), DVDMS alone (DVDMS), ultrasound alone-30s (U-30s), ultrasound alone-60s (U-60s), ultrasound alone-90s (U-90s), sonication-30s plus DVDMS (S-30s), sonication-60s plus DVDMS (S-60s), and sonication-90s plus DVDMS (S-90s). For the DVDMS and ultrasound plus DVDMS groups, the cells were incubated with 0.05 μM DVDMS for 3 h in the dark.

### Cellular uptake of DVDMS detected by flow cytometry

To determine the intracellular DVDMS quantity, cells were collected after different incubation time points (0, 0.5, 1, 2, 3, 4, 5, 6 h) and detected by flow cytometry (Guava EasyCyte 8HT; Millipore Corporation, Billerica, MA, USA). The mean fluorescence intensity of DVDMS was recorded under the same measurement conditions.

### Cellular uptake of four types of porphyrins

The S180 and NIH3T3 cells (8 × 10^4^ cells/well) were seeded into 24-well plates and they were treated by the various concentrations (1 μg/ml, 2 μg/ml and 5 μg/ml) of different sonosensitizers, including DVDMS, protoporphyrinIX (PpIX), hematoporphyrin (Hp) and hermimether (HMME) for 3 h at 37 °C in a CO_2_ incubator. Then the cells were washed twice with PBS and seeded into black 96-well plates (3 × 10^4^ cells/well) prior to imaging using a Xenogen IVIS spectrum system (Xenogen, Alameda, CA, USA). The fluorescence intensity of the cell surface was measured and normalized to photons per second per centimeter squared per steradian (p/s/cm^2^/sr).

### Cell viability assay

The cytotoxicity of SDT was analyzed by using the MTT assay in different types of cells, as previously described^39^. Cell survival was calculated using the following equation:


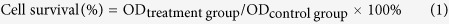


### Analysis of cell apoptosis

The Guava Nexin assay kit (KeyGEN Biotech. Nanjing. China) was used to quantify the number of cells undergoing apoptosis. Annexin V-PE can detect phosphatidylserine on the external membrane of apoptotic cells; the cell-impermeant dye 7-aminoactinomycin D (7-AAD) is used as an indicator of cell membrane integrity. Briefly, 100 μL of cells from each sample was suspended in a mixture of 100 μL Annexin V-PE and (7-AAD) binding buffer and incubated at room temperature for 20 minutes. Samples were detected by flow cytometry (Guava easyCyte 8 HT).

### Endothelial cell capillary-like tube formation assay

The tube formation assay was conducted as described previously^40^. Briefly, each well of a 96-well plate was coated with 60 μL of growth factor reduced-Matrigel (BD Bioscience, San Jose, CA, USA) and incubated at 37 °C for polymerization. Various treatments were implemented prior to cell seeding. Then, the cells were plated into the Matrigel layer at a density of 2.8 × 10^4^ cells per well for 4–6 h. Cells with tube networks were visualized using an Olympus microscope. Three independent experiments were performed.

### *In vivo* S180 xenograft model

The animal experiments were performed in accordance with the National Institute of Health’s Guide for the Care and Use of Laboratory Animals and were approved by the university’s Institutional Animal Care and Use Committee of Shaanxi Normal University (Xi’an, China).

The ICR mice (female) were supplied by the Animal Resource Center of the Fourth Military Medical University (Xi’an, China). The mice were and housed at room temperature with a 12 h light/dark cycle and allowed free access to food and water. After a one week acclimation period, the ICR mice were subcutaneously injected in their right flanks with 0.1 ml of S180 cells (1 × 10^7^ cells/ml). When the tumors reached an average diameter of 5–7 mm, the tumor-bearing mice were randomly assigned to different groups.

Body weight and tumor growth were measured every other day. Tumor growth was determined by measuring the longest (a) and shortest (b) diameter with a caliper, and the tumor volume (V) was calculated according to the following formula:


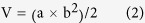


The mice were sacrificed at the end of experiment. Solid tumors and the main organs (heart, liver, spleen, lung and kidney) were excised and weighed. A portion of the tumors and organs were fixed in buffered formalin for histological and immunohistochemical analysis. The tumor weight inhibition ratios were calculated as:


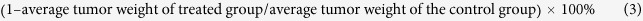


### Histological examination by hematoxylin and eosin (H&E) staining

Tumor tissues and the main organs from different groups were fixed with 10% buffered formalin for 24 h, then paraffin-embedded, sectioned, and stained with hematoxylin and eosin (H&E). Histopathological changes were observed under a light microscope.

### TUNEL assay for apoptotic cells *in vivo*

To identify apoptotic cells *in vivo*, paraffin-embedded tumor tissue sections (6 μm) were stained with a terminal deoxynucleotidyl transferasemediated dUTP nick-end labeling (TUNEL) assay kit (Roche) according to the manufacturer’s instructions. Under the fluorescence microscope, the apoptotic cells exhibited yellow–green fluorescence in the nucleus after excitation with blue light.

### Immunohistochemistry

The paraffin-embedded tumor tissue sections were dewaxed, rehydrated and treated with antigen retrieval in 10 mM citrate buffer (pH 6.0) for 15 min in a microwave-oven. Sections were immersed in 3% hydrogen peroxide solution for 10 min to quench endogenous peroxidase activity. Non-specific binding was prevented by incubation with 5% normal goat serum for 15 min. The sections were incubated with mouse monoclonal anti-PCNA antibody (Abcam, Cambridge, UK), rabbit monoclonal anti-CD34 antibody (Abcam, Cambridge, UK), or anti-vascular endothelial growth factor (VEGF) antibody (Abcam, Cambridge, UK) overnight at 4 °C. Then, antibody binding was detected using a horseradish peroxidase-conjugated secondary antibody (Zhongshan Golden Bridge Biotechnology Co., Beijing, China) for 1 h at 37 °C. The sections were visualized with diaminobenzidine (DAB) solution, lightly counterstained with hematoxylin, and observed using light microscopy.

### Statistical analysis

SPSS 19.0 software (SPSS Inc., Chicago) was used for statistical analysis. All data are expressed as the mean ± standard deviation (SD). Differences among the groups were analyzed using one-way ANOVA. A value of p < 0.05 was considered significant.

## Additional Information

**How to cite this article**: Xiong, W. *et al*. A new sensitizer DVDMS combined with multiple focused ultrasound treatments: an effective antitumor strategy. *Sci. Rep*. **5**, 17485; doi: 10.1038/srep17485 (2015).

## Supplementary Material

Supplementary Table S1

Supplementary Table S2
