# Author Correction: A new sensitizer DVDMS combined with multiple focused ultrasound treatments: an effective antitumor strategy

**DOI:** 10.1038/s41598-021-96653-5

**Published:** 2021-08-23

**Authors:** Wenli Xiong, Pan Wang, Jianmin Hu, Yali Jia, Lijie Wu, Xiyang Chen, Quanhong Liu, Xiaobing Wang

**Affiliations:** Key Laboratory of Medicinal Resources and Natural Pharmaceutical Chemistry, Ministry of Education, National Engineering Laboratory for Resource Developing of Endangered Chinese Crude Drugs in Northwest of China, College of Life Sciences, Shaanxi Normal University, Xi’an, 710062 Shaanxi China

Correction to: *Scientific Reports* 10.1038/srep17485, published online 03 December 2015

This Article contains errors in Figure 7B.

As a result of an error during figure assembly, images corresponding to sample U-2 were used also for samples U-1 and S-1. The correct images for these samples are shown below as Figure 1 and Figure 2.

**Figure 1:**
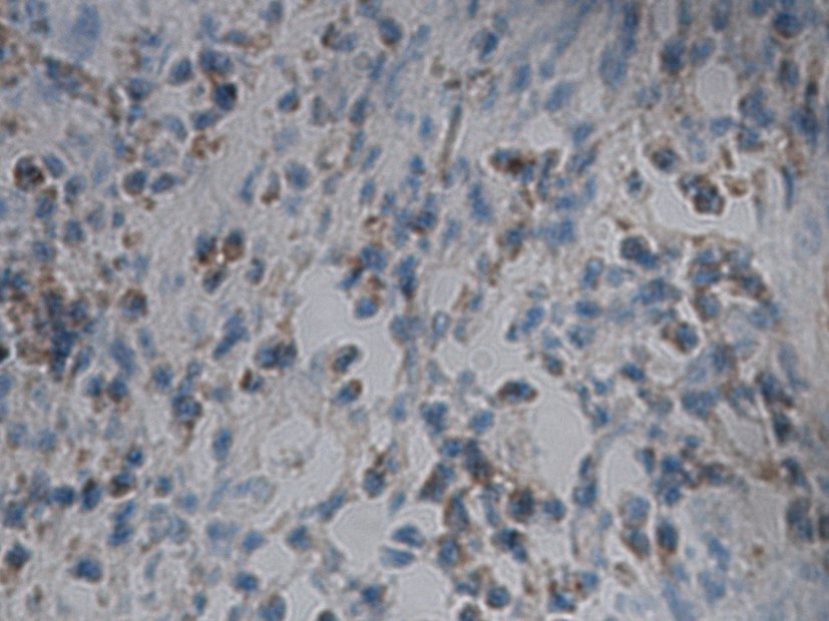
The VEGF level after treatment; sample U-1.

**Figure 2:**
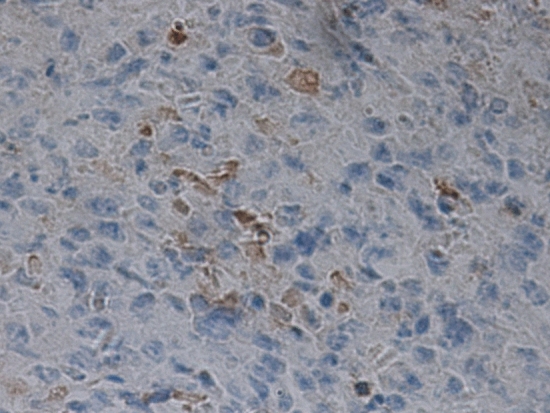
The VEGF level after treatment; sample S-1.

The quantification shown in Figure 7B was done at the time of data acquisition and is therefore unaffected by this error. The conclusions of the Article are not affected by this correction.

