# Supplementary material for: A new sensitizer DVDMS combined with multiple focused ultrasound treatments: an effective antitumor strategy
Source: Sci Rep. 2015 Dec 3;5:17485. doi: 10.1038/srep17485 (PMC4668354; doi:10.1038/srep17485)
Supplement: Supplementary Table S1 [file srep17485-s1.pdf]

Supplementary information (Table 1)

Title: A new sensitizer DVDMS combined with multiple focused ultrasound treatments: an effective antitumor strategy

Authors: Wenli Xiong[1]

Pan Wang[1]

Jianmin Hu[1]

Yali Jia[1]

Lijie Wu[1]

Xiyang Chen[1]

Quanhong Liu[1]

Xiaobing Wang[1]*

Table 1.Tumor weight in each group at the 15th day after treatment

Groups	Tumor weight (g)	Inhibition rate (%)	P value (compared with Control)
Control	5.41 ±0.38		
DVDMS	4.69 ±0.25	19.71	$p < 0.05$
U-1	3.16 ±0.44	32.56	$p < 0.05$
U-2	2.73 ±0.17	47.93	$p < 0.01$
U-3	2.38 ±0.06	56.12	$p < 0.01$
S-1	2.01 ±0.18	59.33	$p < 0.01$
S-2	1.58 ±0.24	72.59	$p < 0.01$
S-3	0.55 ±0.22	89.82	$p < 0.01$
