# Supplementary material for: A new sensitizer DVDMS combined with multiple focused ultrasound treatments: an effective antitumor strategy
Source: Sci Rep. 2015 Dec 3;5:17485. doi: 10.1038/srep17485 (PMC4668354; doi:10.1038/srep17485)
Supplement: Supplementary Table S2 [file srep17485-s2.pdf]

Supplementary information (Table 2)

Title: A new sensitizer DVDMS combined with multiple focused ultrasound treatments: an effective antitumor strategy

Authors: Wenli Xiong[1]

Pan Wang[1]

Jianmin Hu[1]

Yali Jia[1]

Lijie Wu[1]

Xiyang Chen[1]

Quanhong Liu[1]

Xiaobing Wang[1]*

Table 2.The Organs weight were measured at the 15th day after treatment

Groups	Organs weight (g)				
	Heart	Liver	Spleen	Lung	Kidney
Control	0.179±0.024	1.566±0.151	0.171±0.037	0.196±0.017	0.346±0.008
DVDMS	0.173±0.030	1.569±0.119	0.169±0.048	0.192±0.025	0.345±0.032
U-1	0.170±0.023	1.568±0.192	0.173±0.044	0.197±0.017	0.346±0.026
U-2	0.173±0.023	1.573±0.152	0.171±0.053	0.196±0.031	0.342±0.031
U-3	0.177±0.028	1.568±0.069	0.170±0.020	0.197±0.028	0.342±0.015
S-1	0.175±0.023	1.561±0.191	0.171±0.055	0.196±0.025	0.342±0.011
S-2	0.173±0.026	1.515±0.129	0.162±0.016	0.197±0.027	0.388±0.014
S-3	0.175±0.021	1.563±0.074	0.171±0.022	0.196±0.134	0.341±0.013
